# The progress and prospect of sentinel lymph node mapping in endometrial carcinoma

**DOI:** 10.3389/fmed.2023.1306343

**Published:** 2023-11-23

**Authors:** Jiayan Shi, ZhiXian Shi, Qianrun Chen, Ce Bian

**Affiliations:** Department of Gynecology and Obstetrics, Key Laboratory of Birth Defects and Related Diseases of Women and Children, Ministry of Education, West China Second Hospital, Sichuan University, Chengdu, China

**Keywords:** sentinel lymph node, endometrial cancer, lymphadenectomy, sentinel lymph node mapping, algorithm

## Abstract

Sentinel lymph node (SLN) refers to the initial site of the lymphatic drainage from a primary tumor area. Identifying the SLN and analyzing tumor involvement can predict the status of the remaining lymph nodes. Accordingly, sentinel lymph node mapping (SLN mapping) has been brought up and widely applied to cancer therapy for its illuminating role in clinical lymph node resection. Sufficient information to guide surgical pathological staging and adjuvant treatment in endometrial cancer can be rendered by SLN mapping, hence minimizing surgery injury and reducing the incidence of complications. Evidence suggests that using SLN mapping does not affect progression-free survival (PFS) and overall survival (OS) of endometrial cancer patients. Furthermore, there is increasing evidence that using SLN mapping has a high detection rate (DR), sensitivity, and negative predictive value (NPV) for patients with early-stage lower-risk endometrial cancer. This review aims to systematically summarize the advances and application prospects of SLN mapping in endometrial cancer, with an expectation of furnishing reference for the clinical application.

## 1 Introduction

Initially put forward in 1960, sentinel lymph node (SLN) is defined as the first site of lymphatic pathway passing lymphatic metastasis from a primary malignant tumor, which can forecast the involvement of lymph nodes across the drainage area. It has been widely acknowledged that in the lymphatic system, lymph drains away from the primary tumor site in an orderly manner, thus suggesting that the metastatic state of SLN (negative or positive) can indicate the state of subsequent lymph nodes and the metastasis of tumor. More specifically, provided that the metastatic state of SLN is negative, then the ensuing nodes should also be negative. Based on the notion stated above, sentinel lymph node mapping (SLN mapping) as an image-guided procedure to provide ideas for the clinical decision of lymph node resection, has been brought up and widely applied to cancer therapy, such as penile carcinoma, breast cancer and melanoma. As for endometrial cancer, although the application of SLN mapping was raised in 1996 (1), it has only been given unprecedented attention in recent years.

Endometrial cancer is one of the most common malignant tumors of the female reproductive system with rapidly increasing incidence these years (2). According to the National Cancer Institute (NIH), there will be an estimated 65,950 new cases of uterine cancer diagnosed and more than 12,550 deaths in 2022, making uterine cancer the ninth most prevalent cancer in the United States. As stated on the website of NIH, endometrial cancer detected at localized stage has a relatively favorable prognosis, which emphasizes the importance of effective detection, staging methods and appropriate adjuvant treatment. In terms of the staging of endometrial cancer, the clinical staging system of endometrial cancer (I-IV staging) was firstly proposed in 1962. Then in 1988, a surgical-pathological staging system raised by the International Federation of Gynecology and Obstetrics (FIGO) took the place of the surgical staging system. Exact recognition of the lymph node metastatic status is regarded as an important factor in the staging surgery for endometrial cancer since it can implicate prognostic prediction (3). Currently, the standard surgical management is still the mainstay of endometrial cancer treatment, basically covering total hysterectomy, bilateral salpingo-oophorectomy (BSO) and pelvic lymph node dissection (PLND), occasionally coupled with para-aortic lymph node dissection (PALND). However, two large randomized controlled clinical studies all revealed that LND is not beneficial to ameliorate progression free survival (PFS) and overall survival (OS) for patients with early-stage endometrial cancer (4, 5). Therefore, selective lymph node dissection (SLND), generally guided by SLN mapping, provides an alternative to LND by precisely removing a small number of high-quality lymph nodes and minimizing surgical injury without affecting patient prognosis. Conclusively, this review aims to systematically introduce the advances of SLN mapping in endometrial cancer and discuss its application, looking forward to its future development.

## 2 The technique advances of SLN mapping

The most primary objective of SLN mapping is to directly demonstrate the metastatic status of lymph nodes during surgeries in a visible way, hence precisely limiting the dissection of lymph nodes and avoiding systemic lymphadenectomy to the maximum extent. Whether this objective can be achieved or not in endometrial cancer largely depends on the selection of tracer and its injection methods (Table 1).

**Table 1:** Overall results of sentinel lymph node mapping.

Author	N	Injection tracer	Injection site	Overall DR	Bilateral DR	Metastatic nodes	Sensitivity	NPV	FNR
Backes et al. (6)	204	ICG; ISB	Cervical injection	90.2%	83% (ICG); 64% (ISB)	17%	94%	99%	NA
Papadia et al. (7)	42	ICG	Cervical injection	100%	90.5%	23.8%	100%	100%	0%
Biliatis et al. (8)	54	Blue dye	Subserosal injection	85.2%	57.4%	11%	83.3%	97.5%	NA
Mücke et al. (9)	31	Technetium-99 m-nanocolloid	Isthmocervical injection	90.3%	57%	NA	NA	NA	NA
Gorostidi et al. (10)	332	ICG	Cervical+transcervical fundal injection	94.0%	70.5%	16.9%	98.3%	99.6%	1.7%
Geppert et al. (11)	90	ICG	Cervical injection; fundal injection	98% (cervical injection); 80% (fundal injection)	30% (cervical injection); 20% (fundal injection)	19%	NA	NA	NA
Cibula et al. (12)	645	Blue dye	Cervical injection	NA	72%	NA	91%	NA	2.8%

ICG, indocyanine green; ISB, isosulfane blue; DR, detection rate; NPV, negative predictive value; FNR, false negative rate; NA, not applicable.

### 2.1 Injection tracer

Currently, common tracers of SLN mapping include fluorescent dyes, blue dyes, radioactive dyes and carbon nanoparticles. These dyes can be used alone or in combination.

Fluorescent dye indocyanine green (ICG), a safe and effective agent for SLN mapping, has emerged as the most recommended tracer for intraoperative detection of SLN in endometrial cancer owing to its higher sensitivity and specificity compared with conventional tracers (blue dye and radiotracer) (13). In fact, Backes et al. found that in contrast with isosulfane blue (ISB), ICG was conspicuously more effective in detecting SLN (ICG’s detection rate is 83% while ISB’s is 64%) (6). Under the stimulation of near-infrared ray (700–900 nm), fluorescence can be emitted from lymphatic drainage vessels and lymph nodes due to the presence of ICG. And thanks to its low autofluorescence and high tissue penetration, ICG possesses superior signal-to-noise ratio and can show deep-lying lymph nodes, hence making it particularly appropriate for obese patients. Furthermore, Papadia et al. reported that in the application of SLN mapping in high-risk endometrial cancer patients, ICG had acceptable sensitivity, false-negative rate, and negative predictive value as well (7). Yet notably, ICG can result in more interstitial fluid to enter lymphatic channels since it is albumin-bound and causes oncotic pressure. In a consequence, lymphatics can probably and sometimes easily be mistaken for lymph nodes because of their seemingly bigger and swollen appearance, suggesting that surgeons ought to be alert of this pitfall in the application of ICG.

Blue dyes encompass methylene blue, patent blue and isosulfan blue. These dyes can bind to serum proteins following interstitial administration, which can reach peritumoral lymphatic vessels and lymph nodes through lymphatic drainage (14). With no need for advanced imaging system equipment, it merely relies on visual identification of SLNs, which promotes its feasibility (8). Nonetheless, blue dyes’ slow diffusion in lymphatic vessels possibly leads to a lower detection rate. What’s more, the possibility of anaphylactic reactions to patent blue and isosulfan blue during SLN biopsy has been reported, together with the negative effects of intradermal isosulfan dye injection on declined pulse oximetry.

Radioactive tracers, such as technetium-99 ((99 m)Tc) can reach the peritumoral lymph nodes through lymphatic drainage and emits gamma rays with a high concentration in the SLN (15). And its rays can be detected by gamma detector and single-photon emission computed tomography (SPECT–CT) during the operation. It has been reported that the combinative use of technetium-99 m and dyes (ICG or blue dyes) has a remarkable detection rate (16). Despite the reality that Tc(99 m) can penetrate into deep tissue, it still has several drawbacks that cannot be neglected. In particular, its detection depends on special imaging equipment, which results in a higher cost and inconvenience. Moreover, radioactive contamination can potentially occur. All these shortcomings collectively limit its clinical use. But surprisingly, evidence has confirmed that based on Nanotop compound ((99 m)Tc Nanotop), the combination of radiocolloid and ICG is feasible and safe (17).

Carbon nanoparticles (CNPs), a sort of nanosized polymeric carbon granules with an average diameter of 150 nm, are a novel injected suspension (18). Previously, it was mostly used in superficial tumors such as breast cancer and thyroid cancer. As an emerging tracer, it has been applied in tracing lymph nodes and sentinel lymph node detection of endometrial cancer (19). Having been injected into the submucosal layer around the tumor, CNPs can selectively enter lymphatic system due to the interstitial-lymphatic fluid transport effect (18). And since there is a difference in respective permeabilities between lymph and blood systems, CNPs would not permeate into the blood capillaries for its overlarge size, thus implying that CNPs lead to few toxic side effects.

Hybrid tracers, that contain both a radioactive and fluorescent label, were introduced to optimize SLN mapping in endometrial carcinoma (20). Hybrid tracer (ICG-[^99m^Tc]Tc-albumin nanocolloid) combines the benefits of the radiotracer and the fluorescence methods with a single tracer. Injection of ICG-[99mTc]Tc-albumin nanocolloid in 52 endometrial cancer patients, the results showed that hybrid tracer increases the paraaortic detection rate and allows a potential increase in SLN detection (21). Among them, radioactive components allow 97.1% of patients to detect SLN, while fluorescent components detect 80%.

### 2.2 Injection method

While direct peritumoral injection uniformly applies to melanoma, vulvar cancer, and cervical cancer, controversies remain over selecting injection methods for SLN mapping in endometrial cancer due to the complex lymphatic drainage pattern. At present, main injection techniques have been evaluated for SLN mapping in endometrial cancer, including cervical injection, hysteroscopic or transvaginal ultrasound-guided injection and myometrial injection.

Cervical injection has been accepted and recognized by most surgeons for its simple operation without hysteroscopic surgery and high pelvic detection rate (22). In cervical injection, superficial injection can penetrate uterine vessels, isthmus, parametrial, and uterine body, while deep injection can reach para-aortic lymph nodes through pelvic funnel ligament (15). Therefore, a combined superficial (1–3 mm) and deep (1–2 cm) cervical injection is adequate (23). The different options for cervical injection include a 2-sided option (3- and 9-o’clock) and 4-quadrant options (3-,6-, 9-, and 12- o’clock, 2-, 4-, 8- and 10-o’clock). Niikura et al. considered that if lymphatic flow from the uterine cervix was comparable to that from the uterine body in the same patient, then the injection into the cervix would theoretically be more precise (24). Additionally, cervical injection is stable because of the rarity of cervical deformation caused by anatomic variations (such as myomas) and cervical scar from prior procedures (such as conization history or bulky tumor infiltration) (23). However, the probability of missing occult para-aortic lymph nodes is the main argument against the cervical injection. Inspiringly, patients with any site lymph node metastases will receive adjuvant therapy, which theoretically eliminates potential metastatic lesions in para-aortic region (25).

Conceptually, hysteroscopic or transvaginal ultrasound-guided peritumoral injection can directly visualize the tumor and reflect the lymphatic drainage pathway, making them reasonable approaches to detect SLN. Comparing hysteroscopic injection with cervical injection, Ditto et al. have revealed that detection rate of SLN in the para-aortic area was slightly higher in patients receiving hysteroscopic injection (29% vs. 19.5, *p* = 0.18), however, this difference did not reach statistical significance (9). Transvaginal ultrasound-guided myometrial injection of radiotracer exhibited a detection rate of para-aortic SLNs (greater than 45%), together with a high sensitivity (87.5%) and negative predictive value (97.0%) for para-aortic metastases in women with intermediate and high-risk endometrial cancer (26). Both hysteroscopic and transvaginal ultrasound-guided injections were complicated, time-consuming, and technically challenging, hence setting a limitation for a wide acceptance and utilization in real clinical routine. Besides, the potential risk that hysteroscopic injection can cause intraperitoneal dissemination of tumor cells through fallopian tubes has been brought into focus.

In 1996, Burke et al. firstly used sub-serosal myometrium injection of blue dye into the uterine fundus to perform SLN mapping (1). And the detection rate of sub-serosal injection varies from 73 to 95% in recent year’s reports (15). Though this technique is relatively easy to perform, sub-serosal injection has lower sensitivity and overall detection rate.

Apart from those mentioned above, researchers have proposed novel injection methods. Mücke et al. evaluated the clinical feasibility of transcervical subepithelial injection into the isthmocervical region of the myometrium for sentinel detection in endometrial cancer (27). The outcome demonstrated that injection of 10 MBq Technetium-99 m-nanocolloid via isthmocervical myometrium led to high intra-operative detection rates (90.3%), bilateral pelvic detection rates (57%), and para-aortic detection rates (25%). A 6-year single-center prospective study uncovered that the detection rates of dual cervical and fundal indocyanine green injection in endometrial cancer were 94.0% overall for SLNs, 91.3% overall for pelvic SLN, 70.5% for bilateral SLN, 68.1% for paraaortic SLN, and 3.0% for isolated paraaortic SLN (10).

## 3 SLN mapping algorithm

The main goal of SLN mapping is to identify the state of SLN, thereby limiting the need for comprehensive lymphadenectomy (22). To achieve this, it’s a necessity for SLN mapping to have a high bilateral SLN detection rate and a high sensitivity for detection of metastatic lymph nodes, coupled with a low false negative rate. In order to enhance the detection rate and accordingly lower false negative rate, strictly hewing to an appropriate SLN algorithm is of significance. The development of SLN algorithm is based upon the lymph drainage pathways of SLNs in pelvic cavity of endometrial carcinoma patients. Notably, the lymphatic drainage of uterus is considerably complicated. This drainage is presumed to be bilateral since uterus is considered a midline structure (11). There are three channels for pelvic SLN drainage in endometrial cancer: the upper paracervical pathway (UPP), which drains medial external iliac and/or obturator lymph nodes; the lower paracervical pathway (LPP), which drains the internal iliac and/or presacral lymph nodes along the uterine vein; the infundibulo-pelvic pathway (IPP), which drains the para-aortic lymph nodes along the infundibulo-pelvic ligament (28).

It has been reported that the lymph drainage imaging mostly focuses on the UPP pathway. Yet taking the fact that some high-risk patients may have presacral lymph node metastasis into account, the SLN detection of the LPP pathway should not be ignored. In addition, generally, UPP and LPP pathways continuously drain pelvic lymph nodes to the para-aortic lymph node region. Therefore, according to Geppert and his colleagues, tracer-imaging lymph nodes in IPP pathway ought to be regarded as SLN only under the premise that neither in UPP pathway nor LPP pathway can tracer-imaging lymph nodes be found, which helps ensure that the para-aortic lymph nodes taken during surgery are SLN rather than secondary lymph nodes (29). In 2017, Persson et al. (30) first proposed an SLN algorithm to achieve bilateral visualization in UPP and LPP pathways. The bilateral detection rate following reinjection of 96% is the highest reported after ICG use for SLN detection. This algorithm involves both the UPP and LPP pathways to ensure comprehensive detection of bilateral lymph node metastasis. The extent of its implementation depends on the patient’s risk level, as high-risk patients typically require more extensive evaluation, while low-risk patients may only need limited lymph node assessment. This helps better guide preoperative and postoperative treatment decisions. Albeit that the detection effect is excellent, the procedure is cumbersome. And repeated tracer injections conducted in the study may passively affect the detection rate of SLN due to the influence of adjacent lymph node imaging. Subsequently, Bollino et al. (12) optimized the SLN algorithm based on histology and lymphatic anatomy. Specifically, detection of SLN along the UPP and LPP can be restricted to high-risk patients and a full pre-sacral lymphadenectomy should be performed if the LPP pathway cannot be visualized. However, the above SLN algorithm has not been widely applied in the detection of SLN in endometrial cancer, calling for further relevant research.

The current research mostly follows the SLN algorithm put forward in the NCCN guidelines, that is, if bilateral imaging cannot be achieved, the undeveloped lateral lymph nodes should be dissected (31). And suspicious lymph nodes ought to be removed during surgery. Removing para-aortic lymph nodes or not depends on the decision of the surgeon. Strictly following the SLN algorithm can boost the detection rate of SLN and reduce the false negative rate, thereby providing accurate information for clinical decision-making.

## 4 Pathological evaluation and low-volume metastasis

For the pathological evaluation of SLN, the NCCN guidelines recommend the use of pathological ultra-staging. Pathological ultra-staging refers to a combinative assessment approach of both multiple serial sectioning and immunohistochemical staining for surgically removed lymph nodes (32). Not only can this method improve the detection rate of lymph node metastasis, but also identify low volume metastatic disease (LVMD) according to the size of the metastases. In accordance with their diameter, metastases can be divided into macro-metastasis (> 2 mm) and low-volume metastases (LVM) (< 2 mm). And LVM can be further subdivided into isolated tumor cells (ITCs) (< 0.2 mm) and micro-metastasis (MM) (0.2–2 mm) (33). Pathological ultra-staging improves the detection of lymph node micro-metastases in endometrial cancer and can assess the staging of endometrial cancer patients (34, 35). The NCCN guidelines also affirmed the potential value of pathological ultra-staging to detect endometrial cancer low-volume metastases (35). However, pathological ultra-staging takes a considerable consumption of time and requires experienced pathologists to operate, which is not conducive to guiding intraoperative decision-making. And the specific implementation process and application indications need further study.

The clinical significance and management of LVM remain controversial. According to the 2021 NCCN guidelines, LVM is not a basis for staging upgrade, but LVM can guide the formulation of adjuvant therapy (36–38). Studies have found that patients with LVM frequently received adjuvant chemotherapy and had improved oncologic outcomes in comparison to those with macro-metastasis to the lymph nodes, however, low-risk patients with LVM have limited benefit from adjuvant therapy (39). Additionally, there is a possibility of overtreatment in LVM guided adjuvant chemotherapy (40). Therefore, adjuvant therapy should be formulated based on histopathological findings, uterine status, and the overall situation of the patient. In conclusion, at present, the management of LVM patients in clinical practice should be individualized based on the specific situation of patients.

## 5 The application of SLN mapping in endometrial cancer

### 5.1 SLN mapping in low-risk endometrial cancer

The lesions of low-risk patients are mostly confined to uterine corpus, with a low risk of lymph node metastasis. And the ESMO-ESGO-ESTRO proposed that LND is not recommended for low-risk patients (histological grade 1 or 2, superficial myometrial invasion <50%) (41). In the 2018 NCCN guidelines, that SLN mapping can be used for surgical staging when endometrial cancer patients have no metastasis or no obvious extrauterine disease (35). Correspondingly, burgeoning evidence has emerged to support the extraordinary potential of SLN mapping in low-risk endometrial cancer treatment (42). For instance, a FIRES trial investigated 385 patients with stage I endometrial cancer, eventually concluding that the detection rate and negative predictive value of SLN were 86 and 99.6%, respectively (43). Furthermore, study evaluating the cost-effectiveness of three types of lymphadenectomies (systematic LND, selective LND, SLN mapping) in low-risk endometrial cancer found that SLN mapping ultimately outshined other two methods (44). In conclusion, the advantages of low cost and high effectiveness of SLN mapping make it a consensus to replace systematic LND in patients with early-stage and low-risk endometrial cancer.

### 5.2 SLN mapping in high-risk endometrial cancer

Compared with low-risk endometrial cancer, when it comes to high-risk endometrial cancer, the application value of SLN mapping still very controversial. On the one hand, SLN mapping may increase the risk of missed diagnosis of isolated para-aortic lymph node metastases, which appears to be unacceptable in clinical practice (45). Yet on the contrary, SLN mapping in patients with high-risk histologies has shown promising results as a potential alternative to complete lymphadenectomy (46–48). A recent multi-institutional retrospective study concluded that the use of SLN mapping alone, as opposed to SLN mapping with LND in high-risk endometrial cancer, did not impact survival outcomes (49). A recent prospective, multicenter cohort study known as SENTOR-trial investigated the diagnostic accuracy of SLN mapping compared to LND in 156 patients with intermediate- and high-grade endometrial cancer. Among the 27 patients with nodal metastasis, SLN mapping correctly identified 26 of them, demonstrating a sensitivity of 96% (95% CI; 81–100%). This finding supports the conclusion that SLN mapping offers an acceptable level of accuracy in the context of high-grade endometrial cancer (50). More studies have suggested the value of using SLN mapping for surgical staging in high-grade endometrial cancer (51). The application of SLN mapping can shorten the time of laparotomy and laparoscopic surgery as well. Beyond this, the 2022 NCCN guidelines further proposed that SLN mapping can be considered as a method for surgical staging in patients with no extra-uterine metastatic lesion. This suggests that this edition of the NCCN guidelines recognizes the potential application value of SLN mapping in high-risk endometrial cancer patients. More large-scale, multicenter prospective studies are still needed to further settle this controversy.

## 6 Significances of SLN mapping in EC

Numerous clinical studies have confirmed the positive significance of SLN mapping in endometrial cancer. Evidence of the high accuracy and feasibility of SLN mapping has been presented in the retrospective study of Barlin et al. (52). After the application of surgical SLN algorithm recommended in NCCN guideline, the sensitivity could reach 98.1%, together with a clinically acceptably low false negative rate of 1.9%. Furthermore, there is an obvious contrast in the incidence of intraoperative complications such as lymphedema between systematic lymphadenectomy and SLN mapping. According to a perioperative study performed by Geppert et al., the incidence of having lower leg lymphedema in SLN group (1.3%) is astonishingly lower than in the group receiving infrarenal paraaortic and pelvic lymphadenectomy (18.1%) (53). Another noticeable superiority of SLN mapping lies in the reality that it did not compromise the survival prognosis of endometrial cancer patients. In line with a comparative study launched by two Italian institutions, the disease-free survival curves showed a not significant differences between centers and strategies adopted (SLN mapping, SLND, SLN mapping + SLND). What’s more, the difference in the disease-free survival recurrence rates between the two groups remained statistically not significant, when age, stage, grading, histology, lymph vascular space invasion and strategy were considered in a Cox regression analysis. This study also analysis the impact of adjuvant therapy on survival. Adjuvant therapy has an impact on survival on univariate analysis (*p* = 0.002). When the type of adjuvant treatment was added to the multivariate model, the statistical significance was not maintained (*p* = 0.741), whereas stage and grade of disease remained significant (54). Consistently, Eriksson et al. carried out research by, respectively, applying the SLN mapping algorithm and complete pelvic and para-aortic lymphadenectomy, the overall survival and disease-free survival were not significantly different between the two cohorts. But it should be noted that disease-specific survival was significantly different between the two cohorts (*p* = 0.03). Multivariate analyses adjusting for other features was not possible due to the small number of disease-related deaths in both cohorts (0 vs. 5). Adjuvant therapy was administered to 27.1% of patients in the SLN mapping cohort and 10.8% of patients in the SLND cohort (*p* < 0.001) (55). Now more and more studies have proved that SLN mapping alone in high-risk endometrial cancer appears to be an oncologically safe technique over a long observational time. Capozzi et al. compared the long-term survival of high-risk patients who were submitted to SLN biopsy alone versus systematic pelvic lymphadenectomy. Disease-free survival (*p* = 0.74) and overall survival (*p* = 0.62) were not different between the two groups. Furthermore, neither overall survival (*p* = 0.43) nor disease-free survival (*p* = 0.46) was different among these groups in patients with nodal metastasis. This study counted the type of adjuvant therapy, but did not analyse its impact on survival (56). Although growing evidence suggest that SLN mapping in endometrial cancer accurately detects lymph node metastasis. However, prospective randomized trials addressing the oncological outcomes of SLN mapping in endometrial cancer without lymphadenectomy are lacking and many issues are still on research (57).

## 7 Conclusion and perspective

In conclusion, as a guided procedure for the clinical decision of lymph node resection, SLN mapping is cumulatively maturing and its application in endometrial cancer becomes pyramidally promising. Nowadays, ICG is the most recommended tracer, while the cervical injection is favored due to its operational simplicity, reproducibility and high detection rate. Besides, maintaining a low false-negative rate is a priority in any SLN mapping program. However, controversies about the application of SLN detection in patients with endometrial cancer still exist and await to be tackled, such as the standard clinical application of SLN detection in high-risk endometrial cancer, the best algorithm for SLN, and the clinical significance of low-volume metastasis. Therefore, more profound studies are still needed to clarify these extant contentions and more large-scale clinical research is expected to guide the application of SLN mapping in endometrial cancer.

## Author contributions

JS: Writing – original draft, Writing – review & editing, Conceptualization, Investigation. ZS: Writing – original draft. QC: Writing – original draft. CB: Funding acquisition, Methodology, Resources, Supervision, Writing – review & editing.
